# A randomised controlled pilot and feasibility study of music therapy for improving the quality of life of hospice inpatients

**DOI:** 10.1186/s12904-018-0378-1

**Published:** 2018-11-27

**Authors:** Sam Porter, Tracey McConnell, Lisa Graham-Wisener, Joan Regan, Miriam McKeown, Jenny Kirkwood, Mike Clarke, Evie Gardner, Saskie Dorman, Kerry McGrillen, Joanne Reid

**Affiliations:** 1 Department of Social Sciences and Social Work, Bournemouth University, Bournemouth, England; 2 School of Social Sciences, Education and Social Work, Queen’s University Belfast, Belfast, Northern Ireland; 3 School of Psychology, Queen’s University, Belfast, Northern Ireland; 4 Every Day Harmony Music Therapy, Belfast, Northern Ireland; 5 School of Medicine, Dentistry and Biomedical Sciences, Queen’s University Belfast, Belfast, Northern Ireland; 6 Northern Ireland Clinical Trials Unit, Belfast, Northern Ireland; 7 Poole Hospital NHS Foundation Trust, Poole, England; 8 School of Nursing and Midwifery, Queen’s University Belfast, Belfast, Northern Ireland

**Keywords:** Music therapy, Palliative, Hospice, End-of-life, Quality of life, Pilot, Feasibility

## Abstract

**Background:**

Evidence about the effectiveness of music therapy for improving the quality of life of palliative care patients is positive but weak in terms of risk of bias.

**Methods:**

This study aimed to determine the feasibility of a randomised controlled trial to evaluate the effectiveness of music therapy for improving the quality of life of hospice inpatients, as measured by the McGill Quality of Life questionnaire. Objectives included recruitment of 52 participants over 12 months and provision of data to support the calculation of the required sample size for a definitive randomised trial, taking into account the retention rates of recruited participants; and evaluation of the viability of the intervention and the acceptability of the assessment tool. The design was a single-centre, researcher-blinded randomised pilot and feasibility study involving two parallel groups. Participants were recruited from one inpatient hospice unit in Northern Ireland. Eligibility criteria were an Eastern Cooperative Oncology Group performance status of two or lower and an Abbreviated Mental Test score of seven or more. Consenting patients were randomly allocated to the intervention or control group using a 1:1 allocation ratio. The intervention group received up to six individual music therapy sessions over 3 weeks in addition to usual care. The control group received usual care only.

**Results:**

Fifty one participants were recruited over 12 months. Twenty five were allocated to the intervention group and 26 to the control group. Seventy one percent of participants were lost to follow up by week 3, the proposed primary endpoint. The primary endpoint was moved from week 3, when 71% were lost to follow up to week 1, when 33% were lost. The McGill Quality of Life questionnaire was generally acceptable to participants. In order to detect a small to moderate effect size of 0.3, a fully powered study would require the recruitment of 698 participants.

**Conclusions:**

A Phase III randomised controlled trial to evaluate the effectiveness of music therapy in improving the quality of life of hospice inpatients is feasible.

**Trial registration:**

ClinicalTrials.gov: NCT02791048. Registered 6 June 2016.

## Background

Music therapy is defined as the use of music and sounds as part of a developing relationship between the patient and therapist to support and improve physical, mental and spiritual well-being [1]. At its introduction to palliative care in the 1970s, music therapy involved ‘receptive’ approaches where the patient could passively listen to music, and ‘recreative’ approaches where the patient could sing or play previously composed music [2–4]. Since then, it has developed to include improvisational music [5] and song writing [6], both of which involve greater creative input from the patient.

The effectiveness of music therapy in ameliorating a wide range of psychological and physical problems associated with palliative care has been reported in the literature [7]. This includes lowering levels of stress and anxiety, and improving mood, relaxation, overall wellbeing and attitude to life, along with reducing pain levels [8–10]. However, while several studies have suggested that music therapy may improve the quality of life of palliative care patients, many of them had a high risk of bias [10]. As a consequence, evidence about the effectiveness of music therapy for improving the quality of life of palliative care patients remains equivocal and this is an area of important uncertainty for the care of these patients. It has been suggested that this lack of robust evidence can at least partially explain why, while the availability of music therapy for palliative and end-of-life care patients is increasing, funding for it tends to be inconsistent [11].

There are a number of practical considerations that explain the lack of robust randomised trials in this area. Firstly, there are inherent challenges, such as high levels of attrition as a result of the deaths of participants, to conducting randomised trials with palliative care populations [12, 13]. Secondly, challenges such as clinical gatekeepers’ lack of engagement with what they may see as a marginal ‘complementary’ therapy present additional difficulties to those engaging in experimental approaches to music therapy research [14].

### Aims and objectives

This study aimed to determine the feasibility of a randomised controlled trial evaluating the potential effectiveness of music therapy for improving the quality of life of palliative care patients, and to pilot trial procedures. The study objectives were to:

- Provide data to support the calculation of the required sample size for a definitive randomised trial.
- Evaluate the viability of delivering a 3 week music therapy intervention, taking into account the retention rates of recruited participants over that period.
- Evaluate the acceptability of administering the McGill Quality of Life (MQoL) questionnaire to this population [15].
- Evaluate the potential effectiveness of music therapy for improving quality of life.
- Evaluate the potential effectiveness of music therapy for improving inter-familial communication.
- Identify factors that help or hinder the implementation and sustainability of music therapy within a hospice setting.

Only objectives a-d are reported on in this paper. Objectives e and f, which make up part of the critical realist approach that is being taken to this evaluation [16], are reported separately [17].

## Methods

A more detailed explanation of the study’s procedures can be found in our published protocol [18]. In the following, we include changes to the protocol in response to interim findings.

### Study design

This was a single-centre, researcher-blinded randomised feasibility study involving two parallel groups. The intervention group received individual music therapy in addition to usual care. The control group received usual care only.

### Randomization and masking

Consenting patients were randomly allocated to the intervention or control group using a 1:1 allocation ratio. An independent statistician conducted blocked randomization with randomly permuted block sizes, which was used to fill opaque randomization envelopes. These were stored in the clinical investigator’s locked filing cabinet at the hospice and used in sequence, thereby ensuring allocation concealment up to the point of randomization. To maintain researcher blinding, the treatment allocation was forwarded directly to the music therapist by the clinical investigator.

### Setting

Participants were recruited from an 18-bed specialist palliative care unit in a hospice in Northern Ireland.

### Participants

Patients were screened upon admission to the inpatient hospice unit by the clinical investigator. If they were deemed to have sufficient physical and mental capacity to take part (Eastern Cooperative Oncology Group (ECOG) performance status [19] of two or lower; Abbreviated Mental Test (AMT) Score [20] of seven or more), the clinical investigator provided them with information about the trial, including the participant information sheet. If they consented, she referred them to the researcher for potential recruitment. If, following further discussion with the researcher, the patient consented to participate, they were asked to sign an informed consent form. The researcher then informed the clinical investigator. Once recruited, the clinical investigator allocated patients to groups by opening the opaque envelopes in sequence.

After the first month, we recognised that recruitment was lower than expected. Clinical advice indicated that patients having an ECOG performance status that was worse than the initial exclusion threshold would still be capable of participating in the study, and might benefit from doing so. We therefore amended the protocol to include patients with an ECOG performance status of three or lower.

### Intervention

Patients were randomly assigned to two groups. The control group received usual care, as deemed appropriate by the multidisciplinary hospice team. The experimental group received music therapy in an individual setting, delivered by a trained and registered music therapist, in addition to usual care.

A person-centred and creative music therapy approach which employed a systematic therapeutic process that included assessment, therapy and evaluation was adopted, guided by each patient’s needs, interests, preferences and energy levels, and adapted accordingly in the moment [21]. During sessions, an interactive music therapeutic approach was used, whereby the client would engage by singing, playing or listening to known music, or extemporaneously create a melody, rhythm, song or instrumental piece with the therapist’s support. The specific approach used in sessions was agreed with each participant.

Patients could choose to have a carer or significant other present during the intervention. If patients and carers wished, carers could be actively involved in the music therapy sessions.

Music therapy was conducted for up to 45 min twice a week over 3 weeks. In addition to standard clinical documentation, the music therapist completed an intervention manual [22] at the end of each session, including details on who chose the music and what strategy was used from the available suite of music therapy interventions (such as music listening, active music-making, improvising, and legacy work).

### Quantitative research instrument

We used the McGill Quality of Life Questionnaire (MQoL) [15], which has been reported to have the best clinimetric quality rating, content validity, construct validity and internal consistency in a review of quality of life questionnaires for use in palliative care [23]. The MQoL is a 16 item questionnaire which is divided into five sub-measures with a varying number of questions for each sub-measure. The sub-measures are physical symptoms (3 questions), physical well-being (1), psychologic well-being (4), existential well-being (6), and support (2), with each question being scored from zero to ten by the patient. The final score is calculated as one fifth of the mean of the mean scores from each sub-measure. The higher the score, the better the quality of life of the patient and it has been suggested that a difference of 1 to 2 points in the overall score is equivalent to the difference between an average and a good day, and between a bad and an average day [24].

We also collected self-reported baseline data on the patients, including age, gender, ethnicity, marital status and their use of complementary therapies.

### Analysis of outcomes

- The capacity to recruit 52 patients to the study in a recruitment period of 12 months and to retain 70% of these to completion was recorded. 52 participants is a third more than the minimum recommended for good practice in a feasibility study [25] and a 30% attrition allowance is recommended for a palliative care population [26]. These figures, along with input from practitioners and carers on what would be a clinically meaningful difference, were then used to support the calculation of the required sample size for a definitive RCT.
- The viability of delivering a 3 week music therapy intervention was determined by attrition figures along with reasons for attrition.
- The acceptability of the MQoL questionnaire was evaluated at each data collection point by the researcher, who requested verbal feedback from all patients in relation to the acceptability of the questionnaire’s content and level of burden to complete. This verbal feedback was recorded and thematically analysed for any patterns in relation to acceptability. We also monitored and analysed the number of completed questionnaires along with reasons for non-completion using descriptive statistics.
- Our initial intention was to assess potential effectiveness by determining the change over time in the MQoL, which was to be measured at baseline, at 3 weeks (i.e. after completion of music therapy) and 5 weeks (i.e. 2 weeks after completion of music therapy). However, it became apparent that many patients were not surviving long enough after recruitment to reach the 3 week time point and we therefore instigated a MQoL follow-up at 1 week, making the change from baseline to this time point the primary outcome for the effectiveness assessment in this feasibility study. In the protocol, we had envisaged using analysis of covariance to compare the change in quality of life outcomes between the two randomised groups over time. However, for simplicity and given the high attrition rates beyond the first week, we report here the unadjusted mean differences between the groups for the change from baseline to week 1. Results are shown with their associated standard deviations (SD) or 95% confidence intervals (CI). In the absence of a formal power calculation, we advise caution when interpreting our results.

### Reporting

The reporting of this study follows the CONSORT extensions for abstracts and for pilot and feasibility trials.

### Monitoring adverse events

A Serious adverse events (SAE) form was included in each patient’s case report form. As per protocol, any SAE was to be reported to the Principle Investigator within 24 h, and reviewed by the Trial Steering Committee (TSC) at regular intervals throughout the trial. No adverse events were reported during the trial.

### Ethics approval and consent to participate

Ethical approval was provided from the Office of Research Ethics Committee Northern Ireland (ORECNI) (reference number 16/NI/0058), which also provided approval for alterations to the protocol made during the study. Patients and staff provided written informed consent prior to participation in the study.

## Results

### Recruitment, retention and sample size calculation

We recruited and randomised 51 patients between June 2016 and June 2017 (the predesignated time point for ending recruitment), which was 98% of the original target of 52 participants. 43.2% of 118 patients deemed eligible for the study were recruited. Reasons for non-recruitment included those who declined (*n* = 28), early discharge (*n* = 12), non-availability of music therapist due to sickness/annual leave (*n* = 24), and music therapist at full capacity (*n* = 2).

One patient did not complete the baseline MQoL. The first nine patients (music therapy: 4; control: 5) were recruited before we introduced the outcome measurement at week 1. The attrition rate for the week 1 outcome in the other 42 patients was 33%. The attrition rates were much higher for outcome measurement at week 3 (71% of 51 patients) and week 5 (92% of 51 patients), primarily due to the death of the patient or because they were too unwell to complete the MQoL.

### Loss to follow-up

**Fig. 1:**
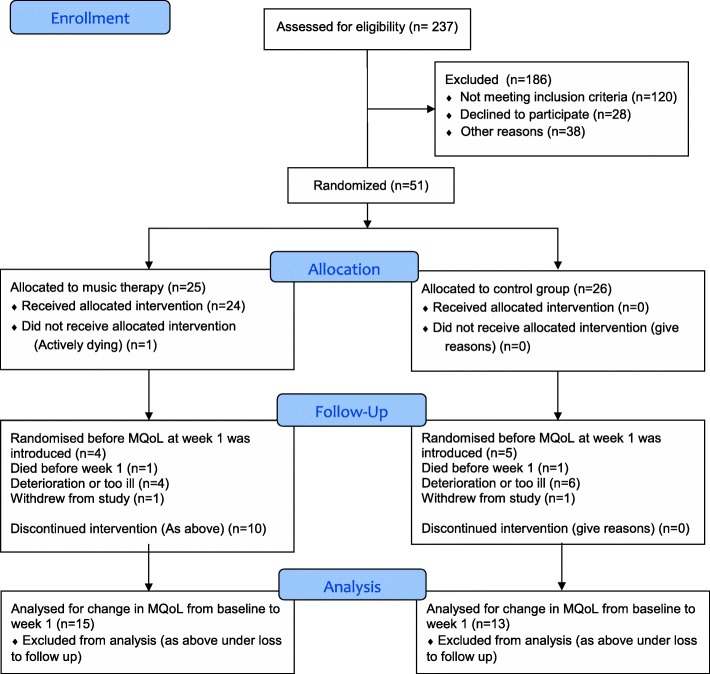
Patient flow through the study

We compared the baseline MQoL data for patients who did and did not complete the MQoL at week 1, to explore any differences between patients who were retained and those who were lost. Visual inspection of the data showed no differences between the randomised groups in the MQoL as a whole or within any of its sub-measures. Reasons for loss to follow-up are shown in the Consort Flow Diagram (Fig. 1). Table 1 shows the baseline characteristics of the randomised patients.

**Table 1:** Baseline characteristics of the randomised patients

	Music therapy (a)	Control (a)	Total (a)	Music therapy (b)	Control (b)	Total (b)
Gender						
Female [n (%)]	20 (80%)	16 (62%)	36 (71%)	16 (76%)	13 (62%)	29 (69%)
Male [n (%)]	5 (20%)	10 (38%)	15 (29%)	5 (31%)	8 (38%)	13 (31%)
Age (years) [mean (SD)]	63 (11.1)	70.7 (10.2)	66.9 (11.2)	63.9 (11.0)	71.3 (10.5)	67.6 (11.3)
Ethnicity						
White [n (%)]	25 (100%)	26 (100%)	51 (100%)	21 (100%)	21 (100%)	51 (100%)
Marital status (missing for one control patient)						
Single [n (%)]	4 (16%)	3 (12%)	7 (14%)	3 (%)	2 (%)	5 (%)
Married/with partner [n (%)]	15 (60%)	15 (58%)	30 (59%)	12 (%)	14 (%)	26 (%)
Divorced [n (%)]	6 (24%)	2 (8%)	8 (16%)	6 (%)	1 (%)	7 (%)
Widowed [n (%)]	0 (0%)	5 (19%)	5 (10%)	0 (0%)	3 (%)	3 (%)
User of other complementary therapies [n (%)]	17 (68%)	10 (38%)	27 (53%)	14 (67%)	7 (33%)	21 (50%)
Baseline MQoLMQoL [mean (SD)]	6.1 (1.2)	6.0^a^ (1.4)	6.1^a^ (1.3)	6.0 (1.3)	6.1^a^ (1.4)	6.0^a^ (1.3)

(a) All 51 recruited patients; (b) 42 patients recruited after introduction of MQoLMQoL at week 1

^a^Excludes one patient who did not complete the baseline MQoLMQ

On the basis of data derived from self-assessed physical wellbeing at baseline, there was little evidence that the lowering of the ECOG score threshold to render patients who were less well eligible for recruitment led to a notable increase in attrition rates. There was no significant correlation between initial rating of physical wellbeing and likelihood of completion.

### Sample size calculation

The difference in improvement between the groups was non-significantly in favour of music therapy: 0.30 (95% CI: - 0.45 to 1.05, *p* = 0.43). Based on a two sample t-test, a sample size of 470 (235 per group) will have 90% power at a two-tailed significance level of 0.05 to detect a mean difference of 0.3 (pooled standard deviation = 1) between groups, representing a small to medium effect size. Taking account of an attrition rate of 33%, a fully powered study would be required to recruit 351 participants per group (see Table 2).

**Table 2:** Change from baseline to week 1 in MQoL and sub-measures

Baseline MQoL, overall and sub-measures [mean, (SD)]	Music therapy (*n* = 15)	Control (*n* = 13)	Mean difference (95% CI)
MQoL overall	0.5 (0.9)	0.2 (1.1)	0.30 (− 0.45, 1.05)
Physical symptoms	1.5 (2.6)	0.4 (3.5)	1.10 (−1.21, 3.41)
Physical well-being	−2.2 (3.8)	−0.2 (3.4)	− 2.00 (−4.67, 0.67)
Psychologic well-being	1.0 (1.6)	0.4 (2.3)	0.60 (−0.89, 2.09)
Existential well-being	1.7 (1.5)	0.0 (2.5)	1.70 (0.14, 3.26)
Support	0.4 (1.9)	0.3 (3.7)	0.10 (−2.13, 2.33)

### Viability of three-week intervention

The study protocol included a plan to recruit from hospice inpatients and outpatients. However, organisational difficulties relating to facilitating outpatients to attend music therapy sessions led to a decision to recruit exclusively from inpatients. One of the consequences of this decision was that the average life expectancy of potential participants was considerably reduced. This was reflected in the afore-mentioned high attrition rates at three and 5 week’s follow-up. This attrition was problematic in relation to therapeutic intent, given that so many patients did not receive the intended dosage of the music therapy, and in assessing the effects of the therapy beyond the first week. We therefore concluded that a three-week intervention was not viable and, as noted above, that the primary outcome should be measured after 1 week. This decision was supported by evidence that a one-week intervention had the potential to be therapeutically effective [27, 28]. Therefore, with approval from the Research Ethics Committee, we amended the protocol during the study to include an additional and earlier follow-up visit after 1 week (after one to two sessions of music therapy) to assess which time-frame is most feasible for an inpatient population at an advanced stage of their illness.

### Acceptability of MQoL questionnaire

Feedback from participants on the acceptability of the MQoL questionnaire was encouraging. Only two participants questioned the pertinence of the information being requested. In terms of burden, only one participant declined to fill out the baseline questionnaire (6% overall). Two patients withdrew from the study. The main issue of concern was the burden that follow-up administration of the questionnaire placed on participants, given the number of people identified who did not fill out questionnaires at follow-up because they were too ill.

### Potential effectiveness

As expected in a feasibility study, the change from baseline to week 1 was not statistically significantly different between the music therapy and the control group. Among the patients who completed both the baseline MQoL and the MQoL at week 1, those in the intervention group (*n* = 15) showed a mean improvement of 0.5 points (SD: 0.9), while those in the control group (*n* = 13) showed a mean improvement of 0.2 points (SD: 1.1). The difference between the groups was non-significantly in favour of music therapy: 0.30 (95% CI: -0.45 to 1.05, *p* = 0.43). Table 2 shows the overall results and the results for the sub-measures in the MQoL. Although most of these are not significantly different between the randomised groups, there was a notable improvement in existential well-being for the music therapy group compared to the control group, and a notable disimprovement in significant disimprovement in physical well-being. However, as noted above these results needs to be interpreted with care and do not provide definitive evidence of a benefit or harm for music therapy in these aspects of patient well-being.

## Discussion

### Recruitment, retention and sample size calculation

While the target sample size was almost attained, the study identified a significant inhibitor to successful recruitment – the unavailability of a music therapist to carry out the intervention, which accounted for 39.4% of those deemed eligible for participation but not recruited. This indicates the need in any future trials for the logistical capacity of music therapy provision to be sufficiently robust to ensure that it is available to all participants who consent and who are randomly allocated to the intervention arm.

The study also identified major problems in relation to retention, with 71% of patients being lost to follow up at week 3 and 92% at week 5; an attrition rate due overwhelmingly to their death or physical deterioration. Clearly, such retention rates indicate that an RCT based on these follow-up time points would not be feasible. In contrast, the attrition rate at week 1 was 33%, marginally above the attrition rate pre-estimation of 30%.

When considered in the context of recruitment rates in this study, the calculation that a fully-powered study would require the recruitment of 702 participants indicates the need for a multi-centre study that includes larger sites. The pilot study recruited 51 participants over 12 months in an 18 bed hospice. However, 26 non-recruitments were due to lack of music therapy resources, which in turn were due to limited funding for the study. A conservative assumption that 50% of those not recruited due to lack of music therapy resources could have been recruited if the adequate resources had been available gives a recruitment estimate of 64 participants per 18 beds per year. We therefore estimate that with access to sites with a total number of 100 beds, and with sufficient resources to ensure the consistent availability of music therapists, the sample size could be achieved over a recruitment period of 24 months.

### Viability of intervention

As noted above, the attrition rates recorded in this study indicate that a three-week intervention is not viable, but a one-week intervention is. However, while the participating music therapists concurred that a one-week intervention consisting of two music therapy sessions was a viable and potentially effective dosage, they reported that much of the first session offered was taken up with introducing the participants to music therapy, the music therapist, and to deciding what mode of music therapy would be most appropriate to their needs. This limited the time available for active therapy. This suggests that the most appropriate regimen would involve three sessions in a week, the first being primarily concerned with introductory activities. While the feasibility of this regimen was not tested in the pilot study, the palliative care clinicians and music therapists involved in the study concurred that, on balance, the extra burden to participants that an extra session involved (which they did not deem to be heavy) would be outweighed by the potential improvement in the effectiveness of the intervention.

### The acceptability of administering the MQoL questionnaire

The main issue in relation to the acceptability of administering the questionnaire related to the fact that 12 out of the 14 patients lost to follow-up at week 1 withdrew because they were too ill to continue and therefore did not complete the follow-up questionnaire. However, given the clinical profile of the patient population involved in this study, this was expected (although the actual attrition rate was 10% higher than the pre-estimated rate). Given their physical deterioration, we do not believe that administering a shorter questionnaire would have had any significant impact on the number of patients completing. Given that the questionnaire was acceptable to the overwhelming majority of participants at baseline, we conclude that the MQoL is an acceptable instrument to use.

### Potential effectiveness

While the study showed a non-statistically significant improvement of the overall scores from baseline to week 1 of the music therapy group compared to the control group, that outcome was the result of the aggregation of divergent scores for the individual domains, ranging from a significant improvement in existential well-being to a significant disimprovement in physical well-being. This disimprovement was a surprising result, not least because the closely related domain of physical symptoms showed a strong improvement. Moreover, a recent systematic review suggested that music therapy may be effective in helping to reduce pain in palliative care patients [10]. Nor have we been able to intuit any putative mechanisms by which music therapy might cause such a response. However, notwithstanding the need to treat this result with caution because of the size of the pilot study, it cannot be ignored. It is therefore important that any future, fully powered study should include both the overall quality of life result and the results of the individual domains as outcomes. Returning to the issue of the burden of the questionnaire, this conclusion underlines the importance of administering the complete questionnaire, rather than ‘cherry-picking’ those domains where improvement might be expected.

### Strengths and weaknesses

The key strength of this study was its ability to demonstrate, in light of the well-documented difficulties of recruiting to music therapy research and the inherent problems of conducting research with a palliative care population, that a large-scale RCT to evaluate the effectiveness of music therapy in palliative care is feasible.

It also clearly demonstrated the weaknesses of the original protocol, most significantly in relation to recruitment and retention. In relation to recruitment, it demonstrated the importance of having sufficient human resources to ensure that everyone who consented to participate would be given the intervention if they were allocated to that group. The crucial role that resources play indicates the need for any future trial to incorporate a robust health economic analysis to ascertain the cost-effectiveness of music therapy compared to standard care.

In terms of retention, the large numbers lost to follow-up at week 3 and beyond demonstrated that a single week intervention, along with the administration of the MQoL at Day 7 after its baseline administration, was the only viable option. However, the use of a 7 day end point for data gathering, while enabling sufficiently powered analysis, would tell us nothing about whether or not music therapy was effective in improving the quality of life of surviving participants in the days and weeks after that point. It is therefore important that a future trial should incorporate evaluation of longer term effects. The difficulty with doing so is the already identified problem of attrition in this patient population, which rose to 71% at 3 weeks, making any statistically significant evaluation of effectiveness at this point unlikely. We therefore propose a compromise endpoint of 14 days at which time the MQoL should be re-administered to consenting patients. This should be supplemented by qualitative data to illuminate the experiences of participants and their close others.

## Conclusions

This study has demonstrated the feasibility of conducting a Phase III RCT to evaluate the effectiveness of music therapy in improving the quality of life of palliative care patients in an inpatient hospice setting. We recommend that the next step for future research is to perform a fully powered trial using a revised protocol that takes into account the findings of this study. We recommend that the primary outcome of the trial should be the difference in change of MQoL from baseline to Day 7 between patient participants who received standard care with music therapy and those who received standard care alone. A secondary outcome should be the difference in change of MQoL between baseline and Day 14.

Robust findings from that trial would make a significant contribution to an evidence base that currently provides commissioners of palliative care services and therapies with equivocal information upon which to base their judgements about the inclusion or otherwise of music therapy in the palliative care armamentarium.

## Abbreviations

AMT: Abbreviated Mental Test
ECOG: Eastern Cooperative Oncology Group
MQoL: McGill Quality of Life Questionnaire
RCT: Randomised controlled trial
